# High-flow nasal oxygen cannula vs. noninvasive mechanical ventilation to prevent reintubation in sepsis: a randomized controlled trial

**DOI:** 10.1186/s13613-021-00963-w

**Published:** 2021-12-13

**Authors:** Radhouane Toumi, Khaoula Meddeb, Mohamed Boussarsar

**Affiliations:** 1 Medical Intensive Care Unit, Farhat Hached University Hospital, Sousse, Tunisia; 2 Research Laboratory N° LR12SP09. Heart Failure, Ibn Al Jazzar Faculty of Medicine, Farhat Hached University Hospital, University of Sousse., Sousse, Tunisia

## Dear editor,

We read with great interest the article of Tongyoo et al. “High-flow nasal oxygen cannula vs. noninvasive mechanical ventilation to prevent reintubation in sepsis: a randomized controlled trial” published in *Annals of Intensive Care* [1]. The authors compared high-flow nasal oxygen cannula (HFNC) and noninvasive mechanical ventilation (NIV) as a ventilatory support in post-extubated sepsis patients seeking a reduction of the reintubation rate using HFNC. A few reservations could be considered.

We felt that the study could have followed a more comprehensive approach concerning acute respiratory failure, pulmonary mechanics and physiology of ventilatory management. This is particularly perceptible in front of the absence of data characterizing patients’ neural drive and pulmonary mechanics, namely, airway pressures, airway collapsibility and respiratory system compliance and resistance [2], both prior and after extubation, which could identify a type of patients benefiting from NIV over HFNC and vice versa. Another important factor to assess, is the patient’s psychological state which could be a cause of extubation failure and thereafter alter the adherence to a type of ventilatory support.

Secondly, the causes of reintubation presented by the authors could mostly be described as consequences of severe acute respiratory failure (hypoxia, inability to clear secretion, altered mental status, cardiac arrest, etc.), whereas it would have been more adapted to characterize the direct clinical causes of the respiratory failure such as, delirium, neuromuscular disorders, laryngeal edema, airways collapsibility, left heart failure, etc.

In our opinion, adapting different means of ventilatory support in accordance to the natural evolution of a disease, or in this particular case, according to the mechanism and severity of the post-extubation acute respiratory failure is more interesting than comparing one technique of ventilatory support to another. NIV is adapted in ARF associated with altered respiratory mechanics while HFNC is adapted to situations at risk of high patient–device interaction generating P-SILI (Patient Self-Inflicted Lung Injury).

Perhaps, taking into consideration the previously stated data, identifying a subgroup of patients presenting the same mechanism of post-extubation respiratory failure that would benefit from one technique rather than the other would be feasible and of great value.

## Data Availability

None.

## Abbreviations

HFNC: High-flow nasal cannula
NIV: Noninvasive ventilation
P-SILI: Patient self-inflicted lung injury
